# High-spatial resolution epidemic surveillance of bacterial meningitis in the African meningitis belt in Burkina Faso

**DOI:** 10.1038/s41598-022-23279-6

**Published:** 2022-11-14

**Authors:** Maxime Woringer, Souleymane Porgho, Christophe Fermanian, Nadège Martiny, Avner Bar-Hen, Judith E. Mueller

**Affiliations:** 1 ENS, École Normale Supérieure, Paris, France; 2 Direction de La Protection de La Santé de La Population, Ministry of Health, Ouagadougou, Burkina Faso; 3 EHESP French School of Public Health, 20 Avenue George Sand, 93210 La Plaine St Denis, Paris, France; 4 Univ. Rennes, EHESP, CNRS, Inserm, Arènes - UMR 6051, RSMS (Recherche sur les Services et Management en Santé) - U 1309, Rennes, France; 5 UMR6282 BIOGEOSCIENCES, University of Burgundy, Dijon, France; 6 Conservatoire National d’arts Et Métiers (CNAM), Paris, France; 7 Institut Pasteur, Paris, France; 8 Present Address: Institut Curie, CNRS UMR3664 & CNRS UMR168, Laboratoire Dynamique du Noyau, PSL Research University, Sorbonne Université, 75005 Paris, France

**Keywords:** Public health, Epidemiology

## Abstract

Despite improved surveillance capacities and WHO recommendations for subdistrict analysis, routine epidemic surveillance of acute bacterial meningitis in the African meningitis belt remains largely limited to the district level. We evaluated the appropriateness and performance of analyses at higher spatial resolution. We used suspected meningitis surveillance data at health centre (HC) resolution from Burkina Faso from 14 health districts spanning years 2004–2014 and analysed them using spatio-temporal statistics and generative models. An operational analysis compared epidemic signals at district and HC-level using weekly incidence thresholds. Eighty-four percent (N = 98/116) of epidemic clusters spanned only one HC-week. Spatial propagation of epidemic clusters was mostly limited to 10–30 km. During the 2004–2009 (with serogroup A meningitis) and 2010–2014 (after serogroup A elimination) period, using weekly HC-level incidence thresholds of 100 and 50 per 100,000 respectively, we found a gain in epidemic detection and timeliness in 9 (41% of total) and 10 (67%), respectively, district years with at least one HC signal. Individual meningitis epidemics expanded little in space, suggesting that a health centre level analysis is most appropriate for epidemic surveillance. Epidemic surveillance could gain in precision and timeliness by higher spatial resolution. The optimal threshold should be defined depending on the current background incidence of bacterial meningitis.

## Introduction

Bacterial meningitis has a case fatality proportion above 10%^1^ and the majority of these cases occur in the African meningitis belt^2,3^. The current understanding of the phenomenon includes a combination of seasonal meningococcal and pneumococcal hyperendemicity related to climatic and environmental factors^4–9^; localized meningococcal epidemics, most likely due to co-factors such as respiratory infection epidemics; and epidemic waves of larger geographic extent, related to meningococcal and pneumococcal strain variations^10–12^. The factors governing localized epidemics emergence and transmission are imperfectly characterized, and more research is needed in order to derive epidemiologic models of the disease.

Starting 2010, a meningococcal serogroup A conjugate vaccine was introduced in meningitis belt countries through mass campaigns^13^, followed by catch-up campaigns and routine infant vaccination. This vaccine introduction appears to have eliminated serogroup A meningitis epidemics, while epidemics due to other serogroups (W, X, C) continue to occur^14,15^ and require vaccine response to limit the epidemic burden. However, a recurrent constraint is that reactive vaccine campaigns often intervene shortly or after the epidemic peak, with limited impact on disease burden. While additional interventions may be relevant, such as antibiotic prophylaxis^16^, an essential piece to improve epidemic response may therefore be accelerated epidemic detection.

We previously have used high resolution surveillance data (i.e., weekly case aggregates by individual health centres, not districts) to investigate risk factors for localized meningitis epidemics^11,12,17^. Similar analyses from Niger before and after serogroup A vaccine have suggested that surveillance and vaccine response at the health centre level could be more effective and efficient than the usual district-level procedures^18,19^. However, moving towards surveillance and vaccine decision in higher spatial resolution is programmatically challenging, as it requires additional resources for updating and training routine procedures. We aim to add supplementary evidence to the advantages of moving to high-resolution surveillance in routine procedures.

Using high-resolution surveillance data from Burkina Faso 2005–2014, before and after introduction of a serogroup A meningococcal conjugate vaccine, we evaluated whether the spatio-temporal structure of bacterial meningitis surveillance data justify high-resolution analyses. In a second step, in an operational analysis, we evaluated whether the use of a simple health centre level incidence threshold for epidemic detection allowed earlier detection and the detection of additional signals compared to district-level surveillance.

## Methods

### Compilation of surveillance data at high spatial and temporal resolution

On a weekly basis, the statistical services of sanitary districts in Burkina Faso collect case reports of suspected meningitis from health centres and aggregate them at the district level. The case definition is based on clinical criteria such as fever and meningeal signs without consideration of laboratory confirmation. Although data are usually presented for the whole district, the weekly health centre data are stored in specific electronic files, which we collected and compiled into a database in collaboration with the Burkina Faso Ministry of Health. As previously described in Woringer et al.^11^, this database covered 14 districts during January 2005–December 2012, nine districts during January 2004–December 2005 and five districts during January 2005–2014. This database reported case numbers per health centre week (HC: the smallest administrative health division in most countries of the meningitis belt and comprises primary health posts, hospitals, and clinics). The database also contained population information at the HC level and was validated against surveillance data that are routinely communicated to WHO with weekly case aggregation at the district level (Supplementary Fig. 1). The study followed the ethical principles of the Declaration of Helsinki (2013 revision^20^). No informed consent had been collected in the context of national routine surveillance, while data were only accessible in form of aggregated case counts. The project received authorisation from national health authorities (Direction de la lutte contre les maladies, Ministry of Health).

The database included 129,342 HC weeks or 2475 HC years, with 15,344 suspected meningitis cases mapped at the HC level. The median radius of a HC was about 11 km and the median area 124 km^2^. The database allowed representing localized epidemic events in individual HC catchment areas during individual weeks, as well as annual epidemic patterns at country level^11^. In particular, the 2006 meningitis season stood out as an epidemic wave in the West subregion, with a high weekly incidence (often exceeding 200 cases per 100,000 inhabitants) and number of epidemic HC weeks.

### Localized epidemics definition and clustering

Firstly, each HC week was classified as a “localized epidemic” (LE) or “non-epidemic”. We determined whether the number of cases in a given HC-week was statistically higher than a given epidemic threshold (e.g., 50, 75 or 100 cases per 100,000 inhabitants; corresponding to binomial test). Compared to a simple threshold analysis, this approach reduced false-positive rates for small populations^11^. We further aggregated these LE into localized epidemic clusters (LEC), assuming that spatially and temporally adjacent localized epidemics (i.e. HC-weeks classified as epidemics using our adjusted threshold) result from the same generative process^21^. To identify such clusters, the adjacency matrices of the yearly shapefiles were derived and epidemic HC-weeks that shared a boundary based on the adjacency matrix and that were zero or one week apart were considered as parts of the same LEC.

### Spatio-temporal analyses

First, we probed the tendency of epidemic health centres for spatial clustering (spatial correlation). To do so, we adopted a spatial point processes approach and derived the spatio-temporal K-Ripley function^22^ of epidemic health centres. The K-Ripley function is the distribution of distances between pairs of health centres identified as localized epidemic (LE): K(r) is the number of pairs of epidemic health centres located at a distance *r* from one another. When individual localized epidemics tend to cluster, a distribution skewed towards short distances is expected; whereas in case of random occurrence, the distribution should follow the law of randomness (i.e., K(r) ∝ πr^2^). To assess the significance of the identified clustering pattern, we derived 95% confidence intervals from resampling under a Null distribution. Distances r at which K(r) exceeds the 95% percentile are interpreted as aggregation into clusters. To account for both spatial and temporal dimensions of our data, we split the time series in several moving windows, such that the K-Ripley function was computed over space on a specified time window (typically 3–4 weeks), leading to the spatio-temporal K-Ripley function, K_ST_(r). This analysis assumes that the spatial and temporal components are independent. The data was analysed separately for the subregions West (districts of Hauts Bassins region and Dédougou district) and North (districts of Nord region). The Boulsa district was excluded from this analysis due to the low number of epidemic events detected and the size of this subregion (Fig. S1). We then used adjacency matrices from the yearly shapefiles for each district to classify each epidemic HC-week as a localized epidemic (LE) or part of a localized epidemic cluster (LEC), with LECs characterized by epidemic HC-weeks that occurred in ≥ 2 adjacent HCs during the same week or ≤ 1 week apart.

Second, to gain some intuition into plausible generative mechanisms of the clustering pattern, we designed three clustering generative processes and denote *E*_*t*_ the number of epidemic HC weeks at time *t* in the empirical database. All three models are conditioned by E_t_, thus accounting for seasonality:

- A fully temporally correlated (TC) model: for the first week of the season *t*, epidemic HC weeks were drawn at random (i.e.: from a Poisson process whose intensity matches the intensity pattern on the region and week of interest). An epidemic HC will remain epidemic until the epidemic starts to vanish at the country-scale in the observed data. More precisely, if the number of epidemic HC increases (E_t_ increases between t = n and t = n + 1) we sample extra HC at t = n + 1. Conversely, if E_n+1_ < E_n_, some HC are removed. Note that there is virtually no spatial clustering in this model (Supplementary Fig. S2a).
- A spatially clustered model (SC): each week was regarded as independent from other weeks and a single cluster comprising of *E*_*t*_ HC was drawn around a random location. This location was changed every week and drawn uniformly within the region of interest. The density of HC followed a normal distribution of standard deviation *σ*, which was the only parameter of this model (Supplementary Fig. S2b).
- A combination of the two models above, incorporating both spatial clustering and temporal correlation (STC). Practically, the STC model was generated similarly as the SC model, but the location of the single cluster of standard deviation *σ* remains the same across the weeks (Supplementary Fig. S2c).

These three models can be seen as a way to investigate whether the temporal correlation or the spatial clustering is the main driver of the enrichment of the spatio-temporal K-Ripley function. Furthermore, this approach can provide clues to build a more sophisticated generative process/mechanism. Similarly, as for the test of spatial clustering, goodness of fit was assessed by derivation of 95% confidence intervals on the simulated spatio-temporal K-Ripley distributions. Although the spatial epidemiology of meningitis is complex and likely involves subtle space–time interactions, the K-Ripley used in this study are aggregated metrics that assume independence between the space and time components.

### Operational analyses

In a second step, we evaluated the use of a simple health centre level incidence threshold for epidemic detection in the period before and after introduction of a serogroup A meningococcal conjugate vaccine. We analysed the number of epidemic signals detected in addition to district-level surveillance and the delay between health centre and district level epidemic signals.

For each health centre, an epidemic week was identified if the weekly incidence was greater or equal to a given cut-off. All consecutive epidemic weeks observed in the same health centre were grouped into one localized epidemic. We assumed that a signal for an epidemic could be detected during the first epidemic week of a localized epidemic. We chose the cut-offs 50, 75 and 100 cases per 100,000 per week based on a method previously described by Tall et al.^21^. Briefly, the weekly incidence cut-offs were evaluated using a receiver-operator-curve, with a reference standard of the 99th and 95th percentiles of annual health centre incidences. At the district level, epidemic signals were identified using the WHO operational threshold of 10 weekly cases per 100,000 (for districts with a population > 30,000)^1^.

Based on laboratory confirmation of suspected cases, conducted within the national surveillance system on a convenience sample of cerebrospinal fluid collected from cases, we defined two surveillance periods: “NmA”, with dominance (or high-level presence) of meningococcal serogroup A, from 2005 to 2009 (2008–09 season); and “other Nm”, with dominance of other serogroups (NmX, NmW, etc.), from 2010 (2009–2010 season) to 2015. These periods approximately correspond to the pre- and post-introduction periods of serogroup A conjugate vaccine.

R software was used to perform spatio-temporal analyses with the packages *stpp*^23^ and *spatstat*^24^, and statistical software Stata/SE 11.2 (StataCorp LP, TX, USA) for operational analysis.

## Results

### Epidemic episodes are spatially and temporally localized

**Figure 1:**
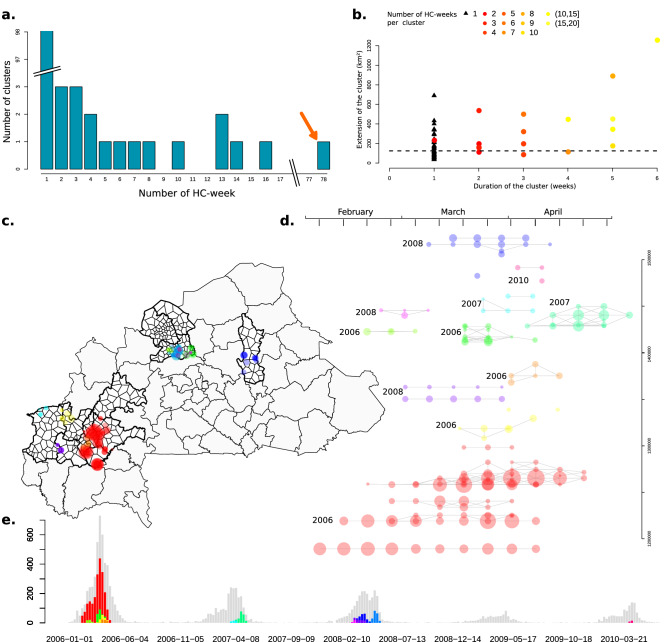
Temporal and spatial dynamics of localized epidemics and localized meningitis epidemic clusters (LEC) in 14 health districts, Burkina Faso, 2004–2014. (**a**) Distribution of the number of epidemic HC-week per epidemic cluster. Orange arrow: 2006 epidemic event. (**b**)Temporal and spatial extension of epidemic clusters. The 2006 epidemic cluster has been omitted for visibility. (**c**) Localization of selected LCE which comprised > 1 HC week. Bold line: borders of the selected districts. (**d**)Temporal and spatial dynamics among LCE comprising > 1 HC week. The clusters are vertically positioned according to geographic latitude and horizontally according to calendar week. The size of the dots represents the number of cases and connected by a line if the HC centroids are less than 14 km apart. (**e**)Temporal occurrence of LCE comprising > 1 HC week. Grey bars: weekly number of cases within the study area. Coloured bars: weekly number of cases per epidemic cluster (stacked).

We identified 292 epidemic HC weeks, which included 3996 (26%, a number consistent with previous studies) of the reported suspected meningitis cases. 194 LE were aggregated into 18 LEC, while 98 of 292 epidemic HC weeks (33.6%) were temporally and spatially isolated, leading to a total of 116 localized epidemics events after clustering (Fig. 1a). When excluding the 2006 epidemic wave (which contained one large LEC spanning 12 weeks and 78 HC weeks with > 1500 cases), most (95%) of the LEC lasted less than 4 weeks and comprised less than six HC weeks. The size of the area impacted by a given LEC was not correlated with its duration (Fig. 1b). With the notable exception of the large 2006 cluster, LEC tended to expand in time rather than space (Fig. 1d–e) and no spatial propagation pattern could be derived (Fig. 1c).

### A spatio-temporal clustering model reproduces the observed epidemic pattern

**Figure 2:**
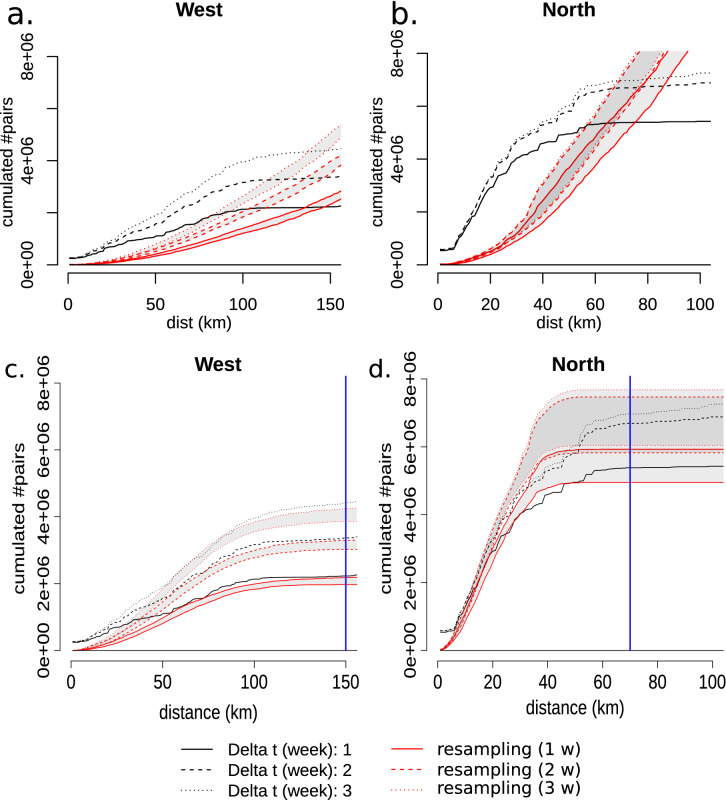
K-Ripley analysis (Null-model) of the meningitis epidemic events for the Western and Northern subregions, in 13 health districts, Burkina Faso, 2004–2014. (**a,c**)Western subregion: Hauts-Bassin *region and Dédougou district. *(**b,d**)Northern subregion: Nord region. Boulsa district was excluded from this analysis due to the low number of epidemic events detected. Black lines, number of pairs of epidemic HC that are closer than a given spatio-temporal horizon on the x-axis: 1, 2 and 3 weeks for spatial horizons ranging from 0 to 100/150 km. Red lines, 5th and 95th percentile of resampled distributions over 20 simula*tions of the generative model. *(**a, b**) Null model without clustering or correlation*. *(**c,d**) Model incorporating both spatial clustering and temporal correlation blue line: range over which the observed K-Ripley is higher than the null models (Western 0–150 km, Northern: 0–70 km).

For spatial scales ranging from 0 to at least 70 km, we found a strong clustering with up to four times more localized epidemics within a given radius than in the Null model (300% enrichment) (Fig. 2a,b). The K-Ripley curves generated by the temporally correlated (TC) model were indistinguishable from the Null model (Fig. S4), suggesting that a model incorporating only temporal correlation but no spatial clustering could not explain the observed pattern. The spatially clustered (SC) model produced K-Ripley curves significantly different from those obtained with observed data, further disproving a model where epidemics occur without temporal correlation (Fig. S4). The STC model including spatial clustering and temporal correlation produced better results than the other two generative models (Fig. 2c,d). The optimized cluster size was 30 km for the West subregion and 10 km for the North subregion. The goodness of fit was highly sensitive to the cluster size (Fig. S3), reinforcing the idea that this parameter is key to describe the spatiotemporal dynamics of localized epidemics.

### Operational analyses at the HC level allow earlier detection of epidemics

**Figure 3:**
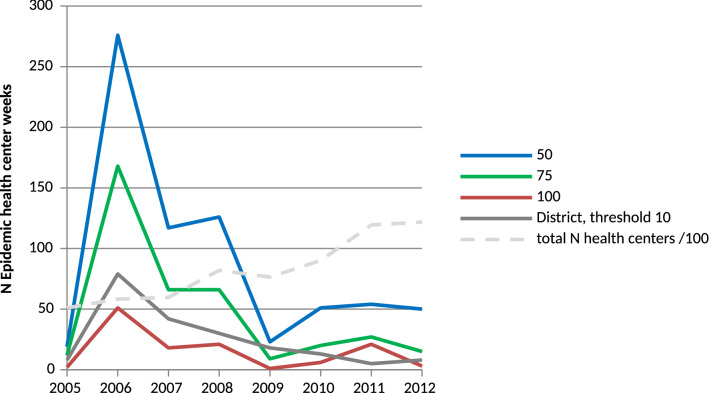
Number of meningitis epidemic weeks detected according to different cut-offs that were applied at the level of health centres (50, 75 or 100 weekly cases per 100,000) or at the level of the district (10 cases per 100,000). 14 health districts in Burkina Faso, 2005–2014. For the periods January–June 2004 and January 2013–December 2014, data from only nine districts were available; these periods therefore were not included in the graph.

Using thresholds of 50, 75 and 100 weekly cases per 100,000, we found 730, 389 and 127, respectively, epidemic health centre weeks and 203 epidemic district weeks using a threshold of 10 per 100,000. After a peak in 2006 (276 weeks at threshold 50 and 79 at district level), the number of epidemic weeks decreased over time, despite an overall increase in the number of available HC-weeks. The decrease was more pronounced with a threshold incidence of 50 or 75 per 100,000, compared to 100; and with a threshold of 10 per 100,000 at the district level (Fig. 3).

For the “NmA” period (2004–2009), using a threshold of 100 cases per 100,000, a localized epidemic was detected in at least one health centre in 22 districts years (Table 1). Four of them (18%) were not accompanied by a signal at the district level. In five of the other 18 district years, the signal was detected earlier at the health centre than the district level. The median delay between health and district signal was 0 weeks (range – 5– + 4). HC level signals after district level signals were due to missing data. Overall in 9 (41%) of the district years with an epidemic event, there was a gain by additional or earlier signal detection when analysing the surveillance data at the HC level.

**Table 1:** Localized meningitis epidemics identified through analysis of surveillance data in health centre level resolution and comparison of the epidemic signals with a surveillance at the district level, by period and by threshold. 14 health centres in Burkina Faso, 2004–2014.

	Years 2004–2009 « NmA »	Years 2010–2014 « other Nm »		
	Cut-off 100	Cut-off 50	Cut-off 75	Cut-off 100
N district years with a localized epidemic in at least one health centre	22	34	15	10
N (%) district years with a localized epidemic but without any signal at the district level	4 (18%)	23 (68%)	7 (47%)	3 (30%)
N (%) district years with localized epidemics and a signal at the district level	18 (82%)	11 (32%)	8 (53%)	7 (70%)
N district years where the signal at the district level was preceded by the signal at the HC level	5	8	3	1
Delay between health centre and district level signal*:				
Mean	− 0.4 weeks	− 2.1 weeks	− 1.4 weeks	0.6 weeks
Median (range)	0 week (− 5–4)	− 1 week (− 9–1)	0 week (− 9–2)	0 week (− 2–4)
N (%) district years with localized epidemic with gain in signal detection using surveillance at the HC level	9 (41%)	31 (91%)	10 (67%)	4 (40%)
Median (range) number of cases that occurred in the HC during the four weeks following the epidemic signal	17 (0–269)	6 (0–219)	2.5 (0–54)	1.5 (0–54)

Cut-offs are expressed as N weekly cases per 100,000 inhabitants.

*negative values mean that the health centre signal preceded the district signal. Positive values usually mean that HC level data were missing for the weeks when the district epidemic was detected.

During the “other Nm” period (2010–2014), using a threshold of 75 cases per 100,000, a localized epidemic was detected in at least one health centre in 15 district years (Table 1). Seven (47%) were not accompanied by a district level signal. In three of the other eight district years, the signal was detected earlier at the health centre than the district level. The median delay between health and district signal was 0 weeks (range – 9– + 2), with the number of cases reported by the HC during the 4 weeks following these signals ranging from 0 to 54. HC level signals after district level signals were due to missing HC data. Overall in 10 (67%) of the district years with an epidemic event, there was a gain by additional or earlier signal detection when analysing the surveillance data at the HC level. This gain increased to 91% of district years with a threshold of 50, which however identified 34 epidemic events. The threshold of 100 produced similar results for the “NmA” and “other Nm” period.

## Discussion

This spatio-temporal analysis of 10-year surveillance data from Burkina Faso at high spatial resolution confirmed that meningitis epidemics appear in a localized manner, rarely propagating to adjacent HC. LEC of more than one HC week represented a minor fraction (16%).

Our operational analysis confirms that surveillance for epidemic meningitis events at health-center level may provide benefits in terms of precision and timeliness. Technical details for this, such as the definition of signal and surveillance entity may need to be adapted to countries’ specificities and the meningococcal epidemiology. The spatial analyses suggest that the ideal grid size would be between 10 and 30 km, which corresponds to health centres in most areas of the meningitis belt. However, the average cluster size between 10 and 30 km is a gross estimate, which needs to be confirmed for other countries in the meningitis belt. Similar results have been presented for meningitis surveillance in Niger^25^ and we suggest that localized meningitis epidemic rarely propagate to neighbouring health centres, if at all. The difference in cluster area between the West and North region may reflect differences in population density (greater in the West region), but also differences in the serogroups causing the observed localized epidemics. The West cluster was dominated by serogroup A epidemics, while serogroup X epidemics occurred in particular in the North region, and these serogroups exhibit distinct spatial dynamics^26^. However, the number of observed epidemics due to non-A serogroups was too small for serogroup-specific analysis. Our results highlight the advantage gained by an analysis of the surveillance data of bacterial meningitis in high-resolution at the level of health centres, particularly for the detection of outbreaks otherwise not observed in district-level surveillance. Although the median time gain relative to signals observed at the district level was not substantial, a large proportion of the signals was not detected at the district level at all.

As suggested by a similar analysis in Niger^18,19^, which in addition evaluated the number of cases avoided and the number of cases per dose of vaccine used, such surveillance at the level of HC could be more effective and efficient than district-level monitoring. As confirmed in our results here, this advantage is even more prominent after elimination of meningococcal serogroup A. This is likely explained by the smaller spatial extent of LE due to serogroups W and X. Mainassara et al. suggested that serogroup C epidemic dynamics in Niger were similar to serogroup A^27^, but that even for serogroup C epidemics, HC surveillance yielded more effective and more efficient reactive vaccine campaigns than district level surveillance^19^.

Analysing meningitis surveillance data at high spatial resolution can also help understanding the factors associated with the occurrence of the localized epidemics. Woringer et al.^11^ found in such analyses that aerosol load was associated with the occurrence of the meningitis season, but not with individual localized epidemics—pointing to the role of additional epidemiogenic factors. Viral infections have been suggested as such co-factors, a hypothesis that was strengthened by Mueller et al.^12^ who found that the occurrence of localized epidemics was strongly associated with high incidence episodes of upper respiratory tract infection notifications. As such, an analysis that would evaluate the role of co-factors, such as respiratory co-infections^12^ or climate-related events such as dust storms^11^, would be of high interest. However, the current dataset does not insure sufficient power to test such co-factors.

We note that most of the cases occur outside epidemic events (74% in our dataset), suggesting that a even a perfect (instant and localized) epidemic response would not be sufficient to avoid cases, and other measures will need to be implemented to control endemic disease. The spatio-temporal K-Ripley analyses we used assumes spatio-temporal independence. The interdependence between values at different locations and timestamp makes formal analytical significance testing difficult, another approach would be to compare our estimates against a null model of zero space–time interaction. Since data is sparse, a space–time model would lead to unstable estimates and a strong loss of power of the tests. Therefore we consider a space–time independent process. Based on data and previous works^25^, one may expect a positive correlation between successive outbreaks therefore our model tends to underestimate the outbreak and lead to conservative tests. We preferred this careful approach in the context of operationability of the proposed results: conclusions can be given for a given distance and/or for a given timelag.

Although we did not estimate the number of cases avoidable through vaccine response in difference surveillance strategies, our results suggest that a simple detection approach at the HC-week level could globally improve the performance and timeliness of epidemic signal detection, and therefore improve the impact of reactive vaccination campaigns. Given that LEC had limited spatial propagation, reactive campaigns could concentrate on epidemic health centres, without need for ring vaccination of the surrounding HC or even the entire district. This would allow better resource allocation for epidemic control.

Few additional resources are required to conduct such surveillance at HC level resolution: with regards to WHO recommendations, no substantial change is required, as the guidelines already recommend the analysis of surveillance data at subdistrict level, for population entities of < 100,000 and < 30,000 inhabitants^1^. Estimates of population per HC would need to be collected and periodically updated, and an additional step needs to be introduced in routine surveillance, consisting in weekly comparison by district officers of the incidences reported from individual HC with pre-defined thresholds. Laboratory analyses to confirm the responsible aetiology or meningococcal serogroups would need to be conducted rapidly for each detected LE signal to guide vaccine response, which now is greatly facilitated by polymerase chain reaction and rapid tests. Procedures for vaccine pre-disposal and clearance for vaccine campaigns would need to be updated to allow more reactive vaccine response. In addition, targeted antibiotic prophylaxis campaigns could be considered^16^. Since 2019, a pilot project in three regions of Niger evaluates the feasibility and effectiveness of routine analysis and compilation of high-resolution surveillance data. We recommend that current and future regional networks of meningitis surveillance consider incorporating procedures for high-resolution surveillance, to foster the development of precision public health for the control of epidemic meningitis in sub-Saharan Africa ^27^.

## Supplementary Information

Supplementary Figures.

## Data Availability

The data that support the findings of this study are available from Direction de lutte contre la maladie (DLM), Ministry of Health of Burkina Faso, but restrictions apply to the availability of these data, which were used under license for the current study, and so are not publicly available. Data related to the present work are however available from the authors upon reasonable request and with permission of DLM, Ministry of Health of Burkina Faso. For this purpose, please contact the corresponding author JEM. The Code to reproduce the results is publicly available on Github (Zenodo https://doi.org/10.5281/zenodo.4925795).
