# Supplementary material for: High-spatial resolution epidemic surveillance of bacterial meningitis in the African meningitis belt in Burkina Faso
Source: Sci Rep. 2022 Nov 14;12:19451. doi: 10.1038/s41598-022-23279-6 (PMC9663584; doi:10.1038/s41598-022-23279-6)
Supplement: Supplementary file 1 — Supplementary Figures. [file 41598_2022_23279_MOESM1_ESM.pdf]

Supplementary Figures for

Woringer et al., High-spatial resolution epidemic surveillance of bacterial meningitis in the African meningitis belt in Burkina Faso; Scientific Reports

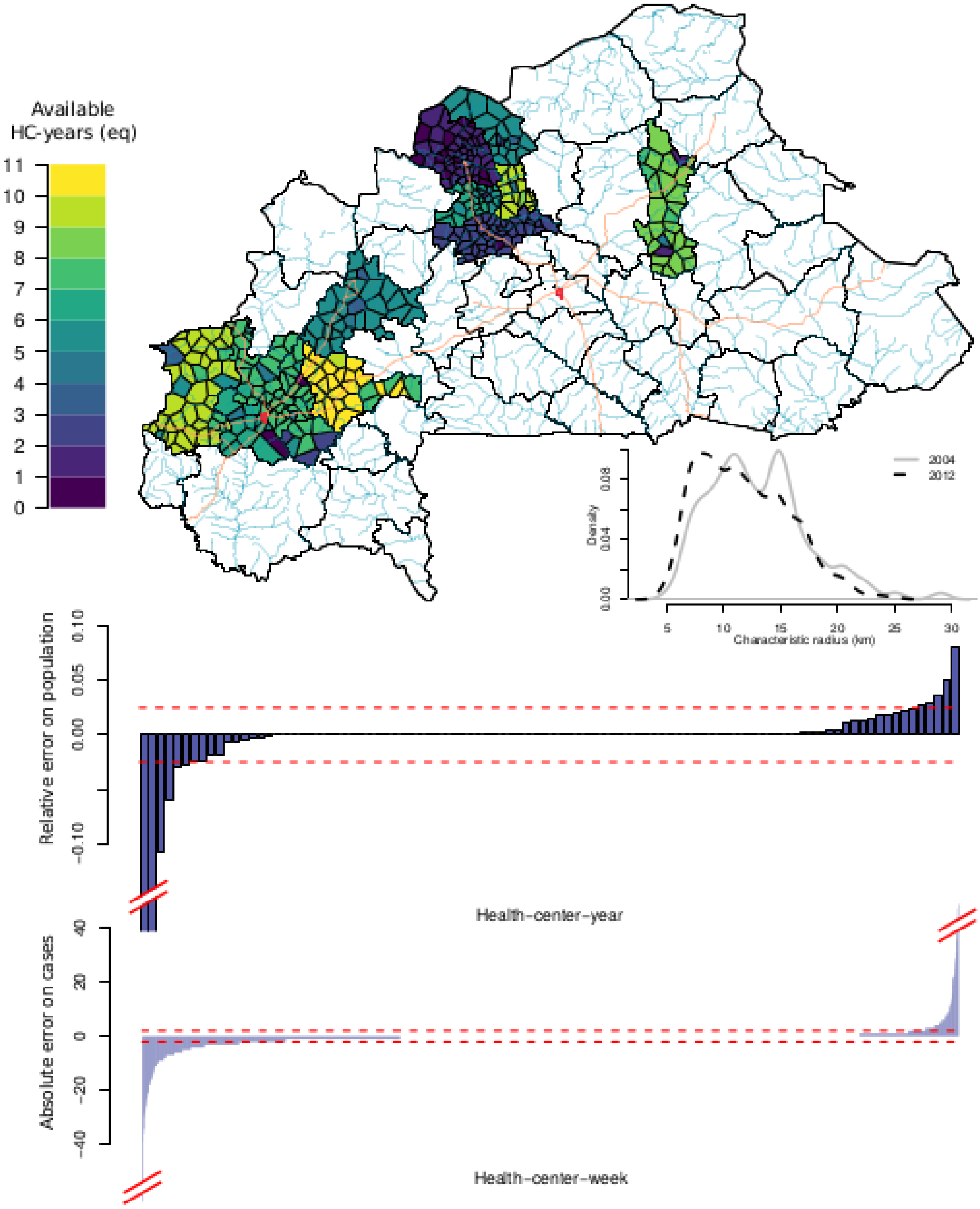

Available
HC-years (eq)
11
10
9
8
7
6
5
4
3
2
1
0
2004
2012
Density
0.00
0.04
0.08
5
10
15
20
25
30
Characteristic radius (km)
Relative error on population
-0.10
0.00
0.05
0.10
Health-center-year
Absolute error on cases
-40
-20
0
20
40
Health-center-week

Figure S1. Validation of the compiled database of suspected bacterial meningitis cases at health center (HC) resolution compared to the dataset at district resolution which is routinely used by the Ministry of Health and the World Health Organization in 14 health districts in Burkina Faso, 2004-2014. **Top panel:** Completeness per HC, expressed as a number of HC available of the period/52. **Middle panel:** Relative error on the population size estimate. For each district year, the population of the corresponding HC are summed and compared to a reference. District years are then sorted by relative error on population. The red, dashed line corresponds to a ±2.5% relative error. **Bottom panel:** Absolute error on the number of cases (assessed by aggregating the data to the district-week level and compared to the WHO reference). The dashed lines correspond to an absolute error of ±2 cases. **Insert:** Distribution of characteristic radii of the HC in 2004 (grey, solid line) and in 2012 (black, dashed line).

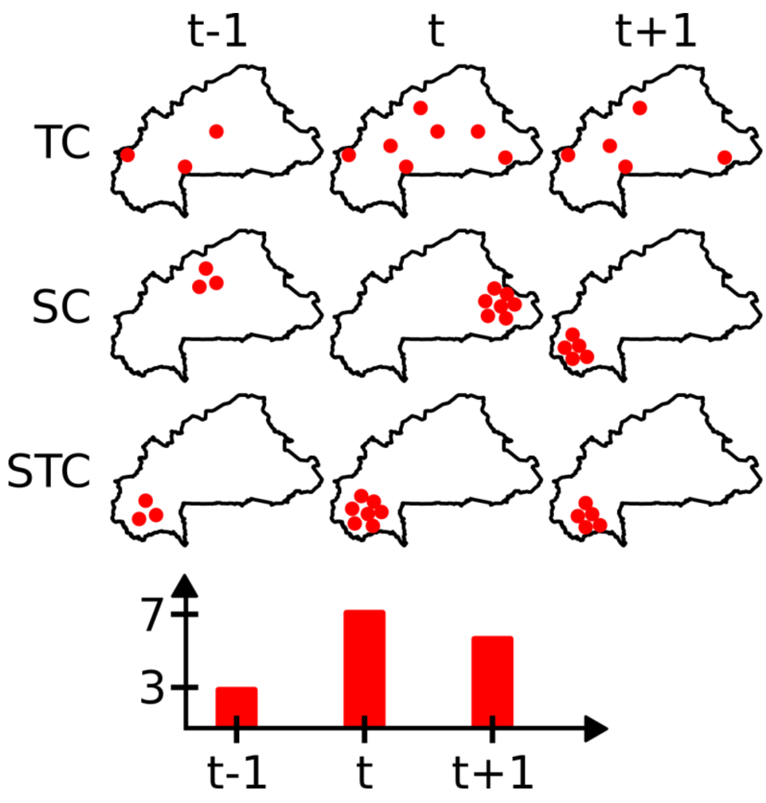

Figure S2.

Illustration of the three generative models. Three consecutive weeks of one epidemic season (t-1, t, t+1) are represented, with 3, 7 and 5 cases, respectively, over the whole region. TC, time-correlated model, SC, spatially-correlated model, STC, model with spatio-temporal correlation.

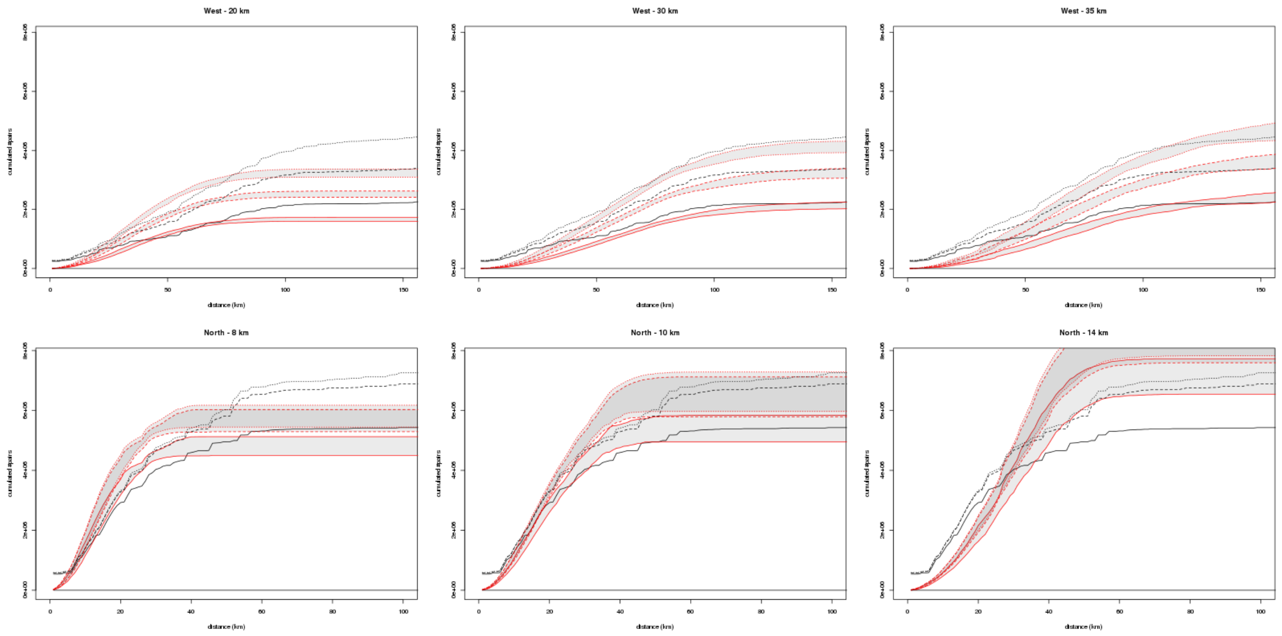

Figure S3. The parameter cluster size is crucial in the STC generative model to reproduce the spatio-temporal K Ripley curves of the tow subregions (West, North).

Top: Western subregion, cluster size of 20, 30 and 35 km (left to right)

Bottom: Northern subregion, cluster size of 8, 10 and 14 km.

The black lines represent the empirical spatio-temporal K-Ripley functions for three temporal horizons (straight line: one week, dashed line: two weeks, dotes line: three weeks) and the corresponding intervals (in red) give the 95% confidence interval over 20 simulations of the generative model. The middle panels show the cluster sizes that were selected as the best fitting models.
